# Scraps

**Published:** 1891-06

**Authors:** 


### SCRAPS

PICKED UP BY THE ASSISTANT-EDITOR.

---

**A Clear Case.**—"When a man dies suddenly without having been attended by a doctor," says a popular guide to the law, "the coroner has to be called in, and an inquest held to ascertain the cause of death. But," adds the writer, "when he dies after having been attended by a doctor, then everybody knows why he died, and an inquest is not necessary."

**Two Views of the Matter.**—Dr. Murdoch Cameron prefaces a paper in the *British Medical Journal* on "The Relief of Labour with Impaction by Abdominal Section" by saying that with the improved methods of operating it is now quite possible in such cases to save both mother and child. And he quotes, as one view of the dreadful alternative formerly presented by craniotomy, the saying of Napoleon, who, when appealed to by Dubois, replied: "Treat the Empress as you would a shopkeeper's wife in the Rue St. Martin; but if one life must be lost, by all means save the mother." Dr. Cameron adds: "In marked contrast to him we had Henry VIII., who, when thus questioned before the birth of his son Edward, exclaimed: 'Save the child by all means, for other wives can be easily found.'"

**Medical Ignorance.**—The *Medical News* says: "The following clipping from the Elk Hart (Indiana) *Review* has been sent to this office: One of our physicians recently received the following letter from a country physician (?): 'Dear dock I hav a pashunt whos phisicol sines shoes that the windpipe has ulcarated of, and his lung have drop intoo his stumick, he is unabel to swoller and I feer his stumick tube is gon. I hav giv hym evry thing without efeckt. his father is welthy Onerable and influenshial. he is an activ membber off the M.E Chirsch and god nose I dont want too loose hym. what shal I due. ans buy returne male. yours in neede.'"

Commenting on this letter, the *British Medical Journal* says: "The diagnosis matches the spelling as perfectly as if by some Leibnitzian *harmonia praestabilita* they had been made for each other. If 'style is the man,' what an ornament to the profession must the author of this edifying document be!"

"**A Surgeon.**"—Bishop Earle, whose "Character" of a physician was given in the *Journal* for March, also drew a picture of the surgeon of his day, whom he described as:—

"One that has ſome buſineſſe about his Building or little houſe of man, whereof Nature is as it were the Tyler, and hee the Playſterer. It is ofter out of reparations, then an old Parſonage, and then he is ſet on worke to patch it againe. Hee deales moſt with broken Commodities, as a broken Head, or a mangled face, and his gaines are very ill got, for he liues by the hurts of the Common-wealth. He differs from a Phyſitian as a ſore do's from a diſeaſe, or the ſicke from thoſe that are not whole, the one diſtempers you within, the other bliſters you without. He complaines of the decay of Valour in theſe daies, and ſighes for that flashing Age of Sword and Buckler; and thinkes the Law againſt Duels, was made meerly to wound his Vocation. Hee had beene long ſince vndone, if the charitie of the Stewes had not relieued him, from whom he ha's his Tribute as duely as the Pope, or a wind-fall ſometimes from a Tauerne, if a quart Pot hit right. The rareneſſe of his cuſtome mak[e]s him pittileſſe when it comes: and he holds a Patient longer than our Courts a Cauſe. Hee tels you what danger you had beene in if he had ſtaide but a minute longer, and though it bee but a prickt finger, hee makes of it much matter. He is a reaſonable cleanely man, conſidering the Scabs hee ha's to deale with, and your fineſt Ladies now and then are beholding to him for their beſt dreſſings. Hee curſes old Gentlewomen, and their charity that mak[e]s his Trade their Almes: but his enuie is neuer ſtir'd so much as when Gentlemen goe ouer to fight vpon Calice Sands, whom hee wishes drown'd ere they come there, rather then the French ſhal get his Cuſtome."

**William Harvey.**—The recent gift of a portrait of William Harvey to the reading-room of the Bristol Medico-Chirurgical Society should serve to remind us how much is to be learned from the example of such a philosophic student. Some points mentioned by one of his biographers may be quoted, and I here condense them:

In 1615, at the age of thirty-seven, Harvey was appointed one of the lecturers at the College of Physicians, and it was in the course of these lectures that he first publicly announced his new doctrines. But though he taught his opinions on this subject *vivâ voce* to his auditors, he continued assiduously to repeat his experiments and verify his observations for many years, before he ventured to commit them to the press. Fabricius ab Aquapendente had taught him, at Padua, that the use of the valves of the veins was to moderate the flow of blood from their trunks into their branches. Harvey more rationally and more obviously insisted that the valves were intended to facilitate the return of the blood to the heart. He demonstrated that the heart being excited to contract by the stimulus of the blood, that fluid is impelled through the arteries, and having served every purpose of secretion and nourishment, returns by the veins to recommence its circulation. Great, however, as was the discovery of Harvey, his doctrine was not so complete and perfect in all its parts as it has since been rendered by the labours of later physiologists. Harvey's work cost him twenty-six years to bring it to maturity. His discovery was ill received; most persons opposed it, others said it was old, very few agreed with him. He had, indeed, his admirers. Witness, for example, certain verses which were addressed "To the Incomparable Dr. Harvey, on his Book of the Motion of the Heart and Blood," in which these lines occur:—

> "There didst thou trace the blood, and first behold
> What dreams mistaken sages coined of old.
> For till thy Pegasus the fountain brake,
> The crimson blood was but a crimson lake,
> Which first from thee did tyde and motion gaine,
> And veins became its channel, not its chaine.
> With Drake and Ca'ndish hence thy bays are curl'd,
> Fam'd circulator of the lesser world."

But the epithet *circulator* in its Latin invidious signification (quack), was applied to him by many in derision, and his researches and discoveries were treated by his adversaries with contempt and reproach. To an intimate friend he himself complained that after his book of the *Circulation* came out he fell considerably in his practice, and it was believed by the vulgar that he was crack-brained. All his contemporary physicians were against his opinion, and envied him the fame he was likely to acquire by his discovery. That reputation he did, however, ultimately enjoy.

About twenty-five years after the publication of his system, it was received in all the universities of the world; and Hobbes has observed that Harvey was the only man, perhaps, who ever lived to see his own doctrine established in his lifetime. Harvey's other great work on the *Generation of Animals* cost him almost as much labour as his work on the *Circulation*.

